# Resistance irrelevant CYP417A2v2 was found degrading insecticide in *Laodelphax striatellus*

**DOI:** 10.1002/ece3.3047

**Published:** 2017-06-02

**Authors:** Mohammad Asaduzzaman Miah, Mohammed Esmail Abdalla Elzaki, Zhaojun Han

**Affiliations:** ^1^ Key Laboratory of Integrated Crop Pest Management in Eastern China (Agricultural Ministry of China) College of Plant Protection Nanjing Agricultural University Nanjing 210095 Jiangsu China

**Keywords:** cytochrome P450 monooxygenases, diagnostic probes, insecticide metabolism, insecticide resistance, *Laodelphax striatellus*, oxidative detoxification

## Abstract

Cytochrome P450 monooxygenases (CYPs) usually overexpressed in resistant strain were found involved in oxidative detoxification of insecticides. In this study, an investigation was conducted to confirm if resistance irrelevant CYPs which were not overexpressed in resistant strain before, were capable of degrading insecticides. Three resistance irrelevant CYPs viz. CYP417A2v2, CYP425A1v2, and CYP4DJ1 from CYP4 family of *Laodelphax striatellus* were randomly selected for experiments. CYP417A2v2 and CYP425A1v2 were found expressed successfully in Sf9 cell line while CYP4DJ1 was not expressed successfully and out of two expressed CYPs, only CYP417A2v2 showed its efficient catalytic activity. For catalytic activity, three traditional model probe substrates and five insecticides were assayed. For the probe substrates screened, *p*‐nitroanisole and ethoxycoumarin were preferentially metabolized by CYP417A2v2 (specific activity 3.76 ± 1.22 and 1.63 ± 0.37 nmol min^−1^ mg protein^−1^, respectively) and they may be potential diagnostic probes for this enzyme. Among insecticides, only imidacloprid was efficiently degraded by CYP417A2v2. Incubation of imidacloprid with CYP417A2v2 of *L. striatellus* and subsequent HPLC, LC‐MS, and MS/MS analysis revealed the formation of imidacloprid metabolites, that is, 4′ or 5′hydroxy‐imidacloprid by hydroxylation. This result implies the exemption of CYPs character that it is not always, all the CYPs degrading insecticides being selected and overexpressed in resistant strains and the degrading CYPs without mutations to upregulate could be candidates during insecticide resistance evolution. This characterization of individual insect CYPs in insecticide degradation can provide insight for better understand of insecticide resistance development.

## 1 INTRODUCTION

The cytochrome P450 monooxygenases (CYPs) are ubiquitous enzymes, constitute a large family, found from lower bacteria to higher mammals and involved in the metabolism of various xenobiotics (Bergé, Feyereisen, & Amichot, 1998). Thus, CYPs are called the most versatile biological catalyst for having the capability of metabolizing structurally different compounds. In insects, CYPs are generally involved in the metabolism of virtually all insecticides, leading to a detoxification (Agosin, 1985; Taylor & Feyereisen, 1996; Wilkinson & Brattsten, 1972). Some time the detoxification mechanism is so active that the insecticide fails to reach to effective level at its molecular target after being metabolized and degraded by these enzymes: such individuals become resistant to insecticides (Taylor & Feyereisen, 1996). CYPs are mainly involved in Phase 1 (Primary) reaction of insecticides metabolism (Feyereisen, 2005; Liu, Li, Gong, Liu, & Li, 2015) and oxidation (hydroxylation) is considered as the most important among phase 1 reactions. In oxidation reaction, a portion of insecticide molecules that enter into insects is transformed to less toxic metabolites and finally excreted (Feyereisen, 2005). Hence, understanding of insecticides degradation through metabolism is crucial for the development of more selective insecticides (Hodgson & Levi, 2010).

The small brown planthopper, *Laodelphax striatellus* (Fallén) (Homoptera: Delphacidae), a notorious phytophagous, causes serious damage directly by feeding grain crops and indirectly by transmitting several plant viruses, that is, rice stripe virus and rice black‐streaked dwarf virus (Kisimoto, 1967). It has a wide distribution range from Southeast Asia to Siberia and to Europe, attacking several important agricultural crops including rice, corn, wheat, oat, and barley (Liu, Zhai, & Liu, 2006). Furthermore, this pest is able to overwinter in the temperate zone of East Asia, like China, and Japan (Matsumura, Otuka, & Watanabe, 2006). In China, *L. striatellus* is considered as a serious pest since the late 1990s and found in all rice‐growing areas. Its density has boosted up dramatically in the beginning of this century, especially in the middle and downstream Yangtze River (the coastal rice production regions of eastern China) and caused great economic damage. Large outbreak of *L. striatellus* (Wei, 2007), serious damage (Liu et al., 2006), and yield loss (Gu, Xue, Shi, & Zhou, 2005) of rice and other crops were commonly occurred incidence in different rice growing areas in china. Usually farmers use insecticides as common practice to suppress *L. striatellus* populations, consequently, this pest develops resistance to various insecticides due to extensive use of chemical insecticides like organophosphate, carbamate, pyrethroid, and neonicotinoid (Wang et al., 2008) as well as cyclodiene organochlorines, phenylpyrazoles, and chitin biosynthesis inhibitors (http://www.pesticideresistance.org). In recent years in China, *L. striatellus* resistance to insecticide has been a frequent incidence and it is field populations have developed variable resistance to different kinds of insecticides (Wang, Zhang, Han, Liu, & Fang, 2010). It is also well documented that field populations of *L. striatellus* developed different levels of (high to extremely high) resistance to imidacloprid, deltamethrin, buprofezin, fipronil, and chlorpyriphos in different areas in China (Gao, Wu, Huang, Mu, & Han, 2008; Ma, Gao, Wei, & Shen, 2007; Wang et al., 2010; Zhang, Chen, Chen, & Yu, 2007). Therefore, insecticide resistance management strategies must be developed to prevent further increase in resistance of *L. striatellus*.

The involvement of various insect CYPs in insecticide detoxification and resistance has been documented for a long time. In the 1990s, several authors reported that different CYPs of *Musca domestica* can metabolize different insecticides, such as aldrin, heptachlor, and diazinon metabolized by CYP6A1 (Andersen 1994; Sabourault et al. 2001), chlorpyrifos, chlorpyrifos oxon, deltamethrin, and cypermethrin were metabolized by CYP6D1 (Hatano & Scott, 1993; Korytko & Scott, 1998; Wheelock & Scott, 1992; Zhang & Scott, 1996) as well as aldrin, heptachlor and diazinon were metabolized by CYP12A1 (Guzov, Unnithan, Chernogolov, & Feyereisen, 1998). Furthermore, several authors demonstrated that imidacloprid was metabolized by *Bemisia tabaci* CYP6CM1vQ (Karunker et al., 2009), *Nilaparvata lugens* CYP6A1 (Ding et al., 2013), and *Drosophila melanogaster* CYP6G1 (Joußen, Heckel, Haas, Schuphan, & Schmidt, 2008). Moreover, it was documented that abamectin is metabolized by CYP392A16 in *Tetranychus urticae* (Riga et al., 2014), pymetrozine is hydroxylated (metabolized) by CYP6CM1 in *B. tabaci* (Nauen, Vontas, Kaussmann, & Wölfel, 2013), pyrethroid (esfenvalerate) is metabolized by CYP9A12 and CYP9A14 in *Saccharomyces cerevisiae* (Yang, Yue, Chen, & Wu, 2008) and by CYP6BQ23 in *Meligethes aeneus* (Zimmer et al., 2014). Some specific CYPs like human CYP3A4 has also been shown to metabolize the imidacloprid (Schulz‐Jander & Casida, 2002). Nonetheless, a biochemical examination on imidacloprid resistant *B. tabaci* was studied by Rauch and Nauen (2003) and showed that enhanced oxidative detoxification of imidacloprid was associated with CYPs. In addition, it was reported that chlorpyrifos resistance was associated with enhanced detoxification and insensitive target enzymes (Wang et al., 2010) and target site mutation conferred fipronil resistance in *L. striatellus* (Nakao et al., 2011).

Different insect CYPs overexpressed in resistant strains or involved in insecticide resistance have been studied and confirmed for their capability of catalyzing insecticide degradation. The CYPs overexpressed in *L. striatellus* resistant to deltamethrin (Xu, Wu, & Han, 2013)*,* buprofezin (Zhang et al., 2012), and imidacloprid (Elzaki et al., 2015) and their capability to degrade insecticides were also studied ((Elzaki et al., 2017) (data published online)). However, little is known about those CYPs that are not overexpressed in any resistant strains of *L. striatellus* and even in any insects before. Therefore, this resistance irrelevant CYPs (not overexpressed, therefore not associated with insecticide resistance) need to be explored for their catalytic activity. Accordingly, in the present work, some gene members from CYP4 subfamily which are well documented for detoxification were randomly selected and functionally recombinant expressed in Sf9 cells and a biochemical investigation was conducted to establish whether resistance irrelevant CYPs in *L. striatellus* are capable of degrading insecticides.

## 2 MATERIALS AND METHODS

### 2.1 Insects

The field population of *L. striatellus* was collected from the paddy field in Jianhu, Jiangsu Province, China, in June 2015 and has been reared since then without any contact with insecticides. All insects were reared on rice seedlings planted in tissue laid (soil less) plastic boxes at 26 (±2)°C under a 12:12‐h light: dark regime at 70%–80% relative humidity.

### 2.2 Insecticide and chemicals

Technical grade imidacloprid (97%), deltamethrin (98%), buprofezin (97%), chlorpyrifos (96.5%), and fipronil (94%) were purchased from Invitrogen Biotechnology Co., Ltd., Shanghai, China. Nicotinamide‐adenine dinucleotide phosphate (NADP^+^), Glucose‐6‐Phosphate (G6P), Glucose‐6‐Phosphate dehydrogenase (G6PDH), and Phenylmethyl sulfonyl fluoride (PMSF) were purchased from Sigma (St Louis, MO, USA). Bovine serum albumin (BSA) was purchased from Fluka (Fluka AG, St Quentin, France). P450 probe substrates *p*‐nitroanisole, ethoxycoumarin, and ethoxyresorufin were purchased from Invitrozen Life Technologies, USA. All other reagents and solvents used throughout the study were of analytical grade unless otherwise stated.

### 2.3 Functional expression of CYP genes in Sf9 cell and microsomal protein isolation

The entire coding region of three CYP genes of *L. striatellus* which were not reported overexpressed in any resistant strain before was obtained from NCBI (http://www.ncbi.nlm.nih.gov), including *CYP417A2v2, CYP425A1v2,* and *CYP4DJ1*. The ORF of each gene, in accordance with the cDNA sequences was amplified by polymerase chain reaction (PCR) using the specific primers designed. For convenient cloning, the restriction sites (underlined) were introduced into the forward and the reverse primers. The forward primer contained a Kozak translation sequence (bolded) and an ATG start codon for proper initiation of translation. Besides, the cytochrome P450 Reductase (*CPR*) was also constructed for the enzyme system to degrade insecticides (Gene accession number of each gene and gene‐specific primers used in this study are listed in Table**** 1).

**Table 1:** Oligonucleotide primer sequences used for cloning *Laodelphax striatellus* CYPs

Gene	Accession Number	Features	Orientation	Sequence (5′–3′)
CYP417A2v2	JX876498	EcoRI	Forward	CGCGAATTC **GCCACC**ATGCTATTGCCTTATCTAC
		XhoI	Reverse	CGCCTCGAGTTATCGTTTCGTCAGTTGAAC
CYP425A1v2	JX876513.1	BamHI	Forward	CGCGGATCC **GCCACC**ATGGGTATTGTATTGGAATATC
		KpnI	Reverse	CGGGGTACCTCATGTGATGGTTTTCTGAATTC
CYP4DJ1	KF422933.1	EcoRI	Forward	CGCGAATTC **GCCACC**ATGTCCACCTGGGGGCTTC
		KpnI	Reverse	CGGGGTACCTCACCTCCTGTGAAATACAATATC
CPR	KJ017971.1	BamHI	Forward	GGATCCATGGAGGTGGAGGCTGAT
		HindIII	Reverse	GACAAGCTT TCAACTCCATACATCGGCAG

After amplification, PCR products of expected size (1470, 1539, 1545, and 2040 bp for *CYP417A2v2, CYP425A1v2, CYP4DJ1,* and *CPR,* respectively) were checked and purified according to the manufacturer's instructions. The purified PCR products were ligated to the pFastBac™ HTA expression vector with T4 DNA ligase (Invitrogen, Carlsbad, USA). The ligation products were transformed into DH10Bac (an *E. coli* strain*)* chemically competent cells (Invitrogen) and plated on the agar plate containing kanamycin, gentamycin, tetracycline, and IPTG according to the manufacturer's instructions. Positive white colonies were used for isolating Bacmid DNA according to the manufacturer's protocol. The successful constructions of recombinant Bacmid DNA were confirmed by PCR (Fig. not shown).

Sf9 Cells were maintained in suspension culture serum‐free (SF‐900 II SFM, Gibco) medium supplemented with 10% (V/V) fetal bovine serum (FBS, Sigma) in the T‐25 flask. The cells were grown in a 5% CO_2_ humidified incubator at 27°C. The cells were used only from a 3 to 4 days old suspension culture in mid‐log phase with a viability >97%. The recombinant baculovirus DNA (Recombinant Bacmid) was transferred into Sf9 insect cells (Gibco) through Bac‐to‐Bac Baculovirus Expression System (Invitrogen) according to the manufacturer's instructions. The titer of the recombinant viruses was determined following the standard protocols of the supplier. Prior to transformation, the cells were plated in a six‐well culture plate and when the cells were at 50%–60% confluence, they were transformed using the Cellfectin reagent (Invitrogen). Sf9 cells transformed by EGFP (Enhanced Green Fluorescent Protein) were used for positive control and untranformed cells for negative control.

Insect cells grown to a density of 2 × 10^6^ cells/ml were coinfected with recombinant baculoviruses containing different CYP genes and CPR with various MOI (multiplicity of infection) ratios to identify the optimal conditions. After 48 hr cells were harvested and washed with PBS, and the microsomes of the membrane fraction were prepared according to standard procedures and stored at 80°C (Phillips and Shephard, 2005). Briefly, the cells were washed twice with 0.1 mol/L, pH 7.8, sodium phosphate buffer and resuspended in precooled lysis buffer (0.1 mol/L, pH 7.8, sodium phosphate buffer, containing 1 mmol/L EDTA, 1 mmol/L DTT, 1 mmol/L PTU, and 1 mmol/L proteinase inhibitor PMSF). The suspension was sonicated in an ice bath and again centrifuged at 10,000 rpm for 10 min. The supernatant was used immediately or kept in −70°C as the enzyme source for checking CYP proteins, catalytic activity, and insecticide metabolism. For detecting the target recombinant CYP protein, 12% SDS–PAGE gels was run on the Bio‐Rad Mini‐Protein II apparatus according to Laemmli (1970) and proteins were visualized by staining with Coomassie Blue.

The expression of functional P450 protein was first estimated by resuspending the microsomes of the membrane in Spectrum Buffer (100 mmol/L Tris‐HCl, pH 7.4, 10 mmol/L CHAPS, 20% (v/v) glycerol, 1 mmol/L EDTA) (Pritchard et al., 1998), adding about 1 mg/ml of sodium dithionite (Na_2_S_2_O_4_) as a reducing agent and recording the absorption spectra (500–400 nm) change after exposing to CO for 1 min. The peak height at 450 nm was used to calculate the P450 concentration (Omura & Sato, 1964). Total protein content was determined by the Bradford method (Bradford, 1976) using bovine albumin as a standard.

### 2.4 Catalytic activity measurements

Different recombinant CYP/CYP microsome preparations were first tested for their P450 monooxygenase activity, with several model probe substrates.

P450 monooxygenase O‐demethylase activities toward the substrates *p*‐nitroanisole (PNOD) was measured in microplates based on the methods from Yang et al. (2004). In brief, 100 μl of 2 mmol/L PNOD solution and 90 μl microsomal proteins were added to each well. The microplate was incubated for 5 min at 30°C and the reaction was initiated by the addition of 10 μl of 9.6 mmol/L NADPH. The absorbance was read with a microplate reader (with a Spectra Max M2 reader, Molecular Devices) at 405 nm and 30°C for 15 min. Reactions were run in opaque 96‐well (flat‐based) plates in triplicate. The activity of CYP417A2v2 microsomes was compared to control microsomes obtained from un‐infected Sf9 cells.

Furthermore, two fluorogenic substrates, that is, ethoxycoumarin (EC) and ethoxyresorufin (ER) were tested for the P450 enzyme activity measurements. Enzyme activity reactions were performed in opaque 96‐well (flat‐based) plates, according to the method described by Burke, Thompson, Weaver, Wolf, & Mayers (1994) with some modifications. Briefly, 5–10 μl of probe substrates were added to each well containing 90–95 μl 50 mmol/L potassium phosphate buffer, pH 7.4 (blank control), 90–95 μl Sf9 cell microsomes (negative control) and 90–95 μl cell microsomes containing 1 pmol of CYP/CPR protein. Total reaction volume was 100 μl. Plates were pre‐warmed for 5 min at 30°C before reactions were initiated by addition of 15 μl 10 mmol/L NADPH to each well. Control reactions in which no NADPH was added were run in parallel. Reactions were run for 30 min before quenching as described by the P450‐Glo kit (Promega). The endpoint signal was then measured by a single tube luminometer (Berthold Detection Systems FB12 Luminometer) and the turnover was calculated.

In the ER assays, the fluorescence was measured by setting the plate reader (Microtitre plate reader, Thermo Labsystems) to 530 nm excitation and 590 nm emission and reading the reaction for 2 min. The results were compared to a standard curve of the O‐dealkylated resorufin fluorophore product. For assaying the EC, the plate was incubated for 30 min at 30°C while shaking. The specific activity of EC‐O‐deethylation was determined using a standard curve. Three replicates of negative control (Sf9 cells) and blank control (PBS) reactions were run for each CYP/substrate combination. Significant differences were determined by one‐tailed T‐test (assuming equal variances).

### 2.5 HPLC analysis of insecticide metabolism

For insecticide metabolism assay, imidacloprid, deltamethrin, buprofezin, chlorpyrifos and fipronil (each in 100 μmol/L conc.) were incubated with recombinant CYP417A2v2/CPR microsomes (0.2 mg/ml total protein content) in 0.1 mol/L potassium phosphate buffer with an NADPH‐regenerating system (Promega; 1.3 mmol/L NADP^+^, 3.3 mmol/L glucose‐6‐phosphate, 3.3 mmol/L MgCl_2_, 0.4 U/mL glucose‐6‐ phosphate dehydrogenase). The total assay volume was 500 μl with three replicates for each data point. Microsomes without NADPH‐regenerating system served as a control. Incubation reactions were carried out at 30°C with 400 rpm orbital shaking and the reactions were stopped at different elapsed time intervals varying from 30 min to 4 hr with 100 ml of acetonitrile and incubated for further 30 min to ensure that all insecticide was dissolved. The quenched reactions were centrifuged at 15,000 rpm for 10 min before transferring the supernatant to glass HPLC vials.

Sixty microliters (60 μl) of the supernatant was injected at a flow rate of 1 ml/min at 30°C. The insecticide and its metabolite was separated on an Acclaim C18 (5, 250, 4.6 mm) reverse phase analytical column (Dionex, USA). Time‐course reactions were run with an isocratic program 80% acetonitrile: 20% water for 15 min. Insecticides elution were monitored by absorption at 270, 230, 245, 290, and 275 nm for imidacloprid, deltamethrin, buprofezin, chlorpyrifos, and fipronil, respectively. The depletion of insecticides was quantified by peak integration (Chromeleon, Dionex).

### 2.6 UPLC–MS analysis and identification of metabolites

The main metabolites of insecticides were identified by UPLC‐MS analysis according to the method described by Karunker et al. (2009). All samples obtained from insecticide metabolism assays were subjected to Ultra Performance LC (Waters Acquity UPLC System, Waters, Eschborn, Germany). Samples were separated by gradient elution using a mobile phase consisting of HPLC water containing 0.1% (w/v) formic acid and acetonitrile 0.1% (w/v) formic acid, with a constant flow rate of 0.6 ml/min. The gradient elution conditions were as follows: 0 min acetonitrile:water 10:90, 1.5 min 95:5, 3 min 95:5, 4 min 10:90, and 5 min 10:90. The mass spectrometer (Agilent Technologies, Inc., Santa Clara, CA, USA) was operated in positive ion mode. High purity nitrogen at 450°C was used as the sheath/auxiliary gas and argon as the collision gas. The capillary temperature was 270°C.

## 3 RESULTS

### 3.1 Functional expression of *L. striatellus* CYPs

**Figure 1:**
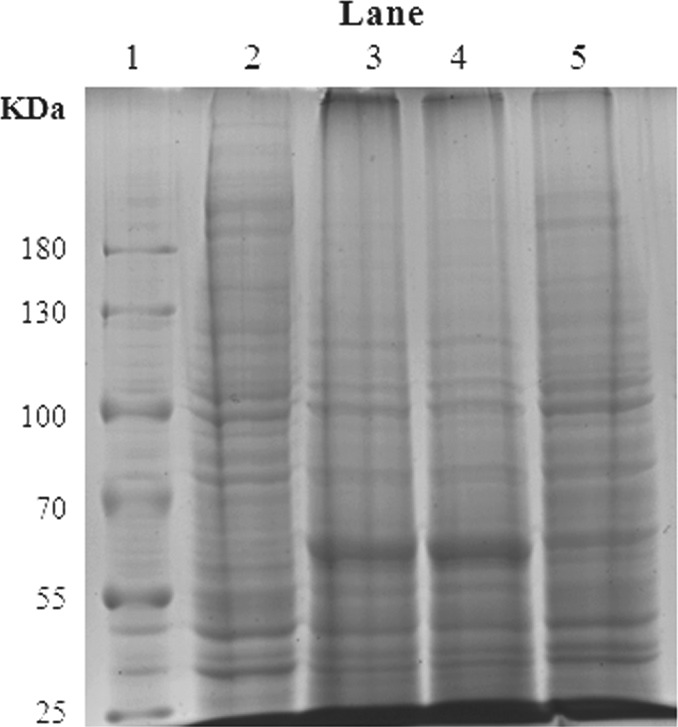
SDS–PAGE analysis of recombinant protein expressed in Sf9 insect cells. Lane 1, Protein molecular weight standards; lane 2, microsomes from baculovirus uninfected Sf9 insect cells (negative control); lane 3, 4, and 5, microsomes from baculovirus infected Sf9 cells treated with CYP417A2v2, CYP425A1v2, and CYP4DJ1 (target recombinant protein, ~60 kDa); the molecular masses of protein standards are shown in the left margin

Three resistance irrelevant CYPs like *CYP417A2v2, CYP425A1v2, and CYP4DJ1* were checked for their functional expression in Sf9 cell lines. The cell microsomes were prepared and subjected to SDS–PAGE analysis. The results showed that *CYP417A2v2* and *CYP425A1v2* were successfully expressed and the distinct band of recombinant proteins with expected molecular weight was identified, whereas there was no characteristic protein band exhibited in the microsomes prepared from Baculovirus infected cells treated with *CYP4DJ1* and uninfected Sf9 insect cells (Figure 1).

**Figure 2:**
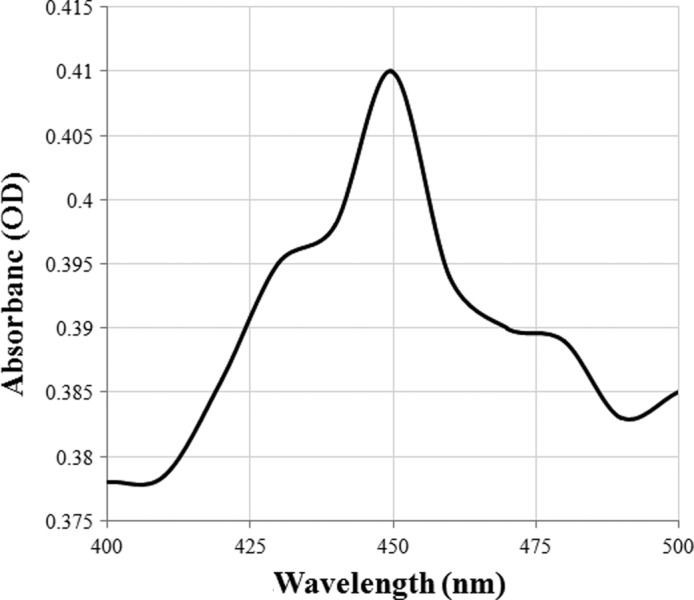
CO‐difference spectra of Sf9 cell microsomes expressing *Laodelphax striatellus* CYP417A2v2

The reduced CO‐difference spectrum was tested in order to confirm the successful expression of intact recombinant CYP proteins in Sf9 cell. The result showed that only the expressed P450 protein of CYP417A2v2 had a characteristic absorption peak at 450 nm, which is the character of the functional P450 proteins (Omura & Sato, 1964) whereas *CYP425A1v2* had no characteristic absorption peak. Thus, CYP417A2v2 protein has been expressed in its P450 form indicating a good‐quality functional enzyme (Figure 2).

### 3.2 CYP417A2v2 catalytic activity against standard P450 model substrates

For checking recombinant CYP417A2v2 for its catalytic activity, three standard P450 model substrates (Fluorescent and chemiluminescent) were first tested, which are routinely used in the pharmaceutical industry (Cohen, Remley, Raunig, & Vaz, 2003) and for diagnostic monitoring of P450 levels for insecticide resistance (Inceoglu et al., 2009). The results demonstrated that the recombinant expressed protein showed good O‐demethylation activity against PNOD with the specific activity 3.76 ± 1.22 nmol min^−1^ mg protein^−1^ as compared with the control Sf9 microsomes. For the fluorescent substrates tested, ethoxycoumarin produced the specific activities (1.63 ± 0.37 nmol min^−1^ mg protein^−1^) while no enzyme activity was detected with the ethoxyresorufin under the assay conditions chosen (Table 2).

**Table 2:** CYP417A2v2 specific activity against standard P450 probe substrates

Substrate (name and structure)		Specific activity (nmol min^−1^ mg protein^−1^)
*p*‐nitroanisole	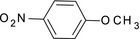	3.76 ± 1.22^a^
Ethoxycoumarin	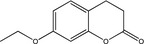	1.63 ± 0.37^a^
Ethoxyresorufin	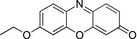	nd^b^

a Results are shown as means ± SE. Significant differences were determined by one tailed T‐tests.

b nd, not detectable.

### 3.3 CYP417A2v2 capability to metabolize insecticides

The metabolism of five insecticides including imidacloprid, deltamethrin, buprofezin, chlorpyrifos, and fipronil were assayed in vitro with CYP417A2v2 microsomes in the presence or absence of NADPH regenerating system. By monitoring of the degradation of parent chemicals and the appearance of metabolites with the reaction time in reverse‐phase HPLC, the enzymatic/catalytic activity of CYP417A2v2 for degradation of different insecticides was determined.

**Figure 3:**
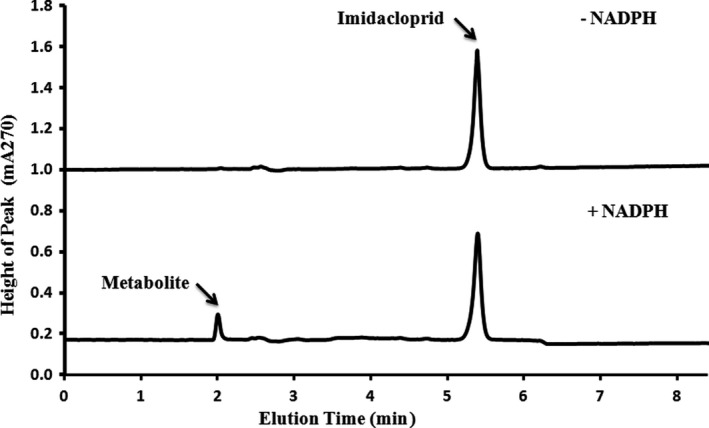
HPLC chromatograph showing NADPH‐dependent metabolism of imidacloprid after 3 hr incubation with microsomes isolated from Sf9 cells recombinantly expressing CYP417A2v2. (Upper Panel) Chromatograph for the CYP417A2v2 reaction mixture in the absence of an NADPH regenerating system (imidacloprid detection; eluted at 5.38 min). (Lower panel) Chromatograph for the CYP417A2v2 reaction mixture in the presence of an NADPH regenerating system (metabolite formation; eluted at 1.99 min and imidacloprid detection; eluted at 5.38 min). Both of the Chromatographs were observed after 3‐hr incubation of reaction mixture

**Figure 4:**
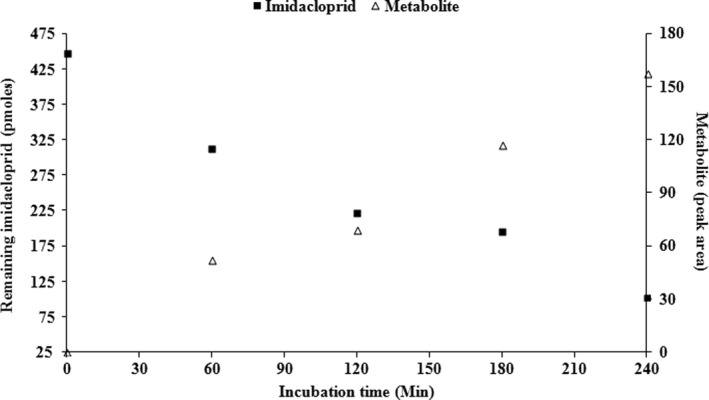
Time course of imidacloprid depletion (■) and the main metabolite formation (∆). Approximately 30% of total imidacloprid was metabolized within 240 min

NADPH‐dependent depletion of imidacloprid (eluting at 5.38 min) and paralleled formation of the main metabolite (eluting at 1.99 min) was observed after incubating the compound with the CYP417A2v2 microsomes (CYP/CPR) in the presence of NADPH regenerating system. In contrast, no change in the control chromatogram of the parental imidacloprid molecule was observed when incubations carried out in the absence of an NADPH regenerating system (Figure 3). The results of monitoring in time course demonstrated that imidacloprid depletion and the metabolite peak formation were time‐dependent (Figure 4). It was observed that about 30% of the total imidacloprid was metabolized within 240 min.

However, CYP417A2v2 was found without any catalytic activity against deltamethrin, buprofezin, chlorpyrifos, and fipronil, as there was no apparent substrate depletion and no obvious substrate turnover observed for any of these compounds.

### 3.4 Identification of insecticide metabolites

HPLC‐MS and MS/MS analysis were employed for identification of imidacloprid metabolites and the results confirmed the generation of a hydroxy‐imidacloprid in the enzyme catalytic reaction stated above.

**Figure 5:**
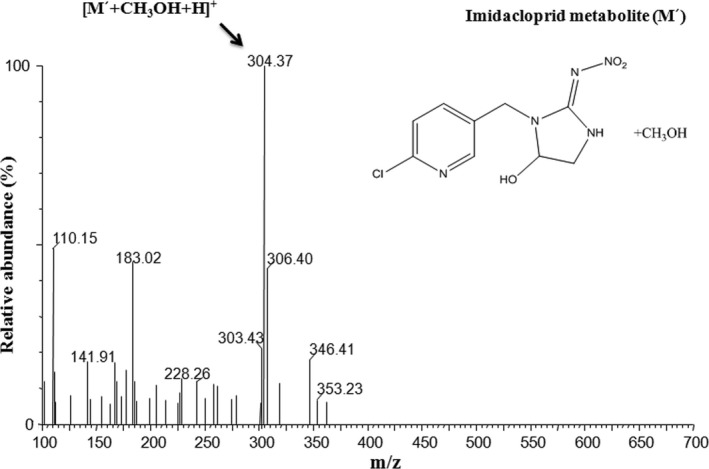
Electrospray ionization mass spectrum of the metabolite hydroxy‐imidacloprid (4′ or 5′ hydroxy‐imidacloprid)

**Figure 6:**
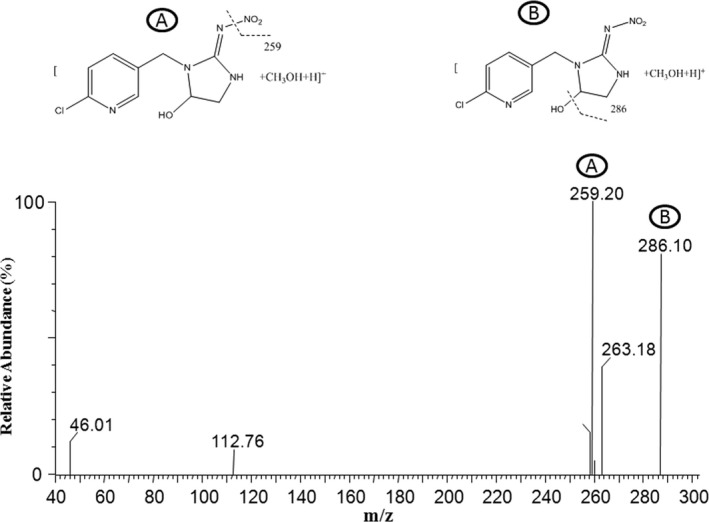
Fragment ion spectrum of the metabolite hydroxy‐imidacloprid. Chemical structures of fragment ions of hydroxy‐imidacloprid are shown

In LC‐MS analysis (Figure 5), the observed molecular ion peak was at m/z [M′+CH_3_OH+H]^+^: 304.01 which denotes 4 or 5‐hydroxy‐imidacloprid. This peak was 16 m/z units higher than the corresponding peak in the spectrum of the parent imidacloprid compound at m/z [M′+H]^+^: 271 that indicates an additional oxygen (Zhang et al., 2007) atom in the metabolite, forming a hydroxyl group. The MS/MS spectrum (Figure 6) of imidacloprid metabolite m/z [M′+CH_3_OH+H]^+^: 304.01 revealed a fragmentation pattern of several ion peaks‐ [259.20]^+^ and [286.10]^+^ that might corresponds to the parent hydroxy‐imidacloprid.

## 4 DISCUSSION

Cytochrome P450 genes (CYPs) are usually overexpressed in resistant strains and involved in insecticide detoxification that ensuing insecticide resistance. Therefore, these CYPs are termed as resistance relevant genes. In this study, three CYPs which were not reported overexpressed before in any resistant strain of *L. striatellus* and not associated with insecticide resistance (resistance irrelevant CYPs) were selected to confirm the catalytic ability for insecticide degradation. As results showed in the SDS‐PAGE analysis, among the three CYPs, CYP417A2v2 and CYP425A1v2 were successfully expressed in Sf9 cells while CYP4DJ1 was not found to be expressed. Not surprisingly, finally only CYP417A2v2 was confirmed for its catalytic activity through a series of biochemical investigation whereas the expressed CYP425A1v2 was not confirmed for its catalytic activity. Assume that several factors could spoil the functional expression in vitro, such as transcriptional factors, post‐translational modifications (N‐glycosylation, in the folding process and the requirement for molecular chaperones to facilitate folding). Thus, the genes had not been successfully expressed did not mean that they are not able to catalyze insecticide degradation. However, the positive result always was thought the proof for the function of the gene. In our study, the resistance irrelevant CYP417A2v2 was found to degrade imidacloprid to its less toxic hydroxy‐imidacloprid through oxidation. This result demonstrated that a resistance irrelevant CYP could be a detoxification enzyme and it is not all the CYPs degrading insecticides being selected in resistant strains.

In the past, resistance associating CYPs were usually thought and proved as the detoxification enzymes, such as *Tetranychus urticae* CYP392A16 could detoxify abamectin (Riga et al., 2014), *B. tabaci* CYP6CM1 hydroxylated pymetrozine (Nauen et al., 2013), CYP9A12 and CYP9A14 of *Helicoverpa armigera* metabolized esfenvalerate (Yang et al., 2008), and *Meligethes aeneus* CYP6BQ23 could hydroxylate deltamethrin and tau‐fluvalinate (Zimmer et al., 2014). Actually, resistance irrelevant CYPs could be detoxification enzymes too and which was really investigated in our study although very little have been studied yet. Firstly, during resistance development, insecticides select only those detoxification enzymes which have been upregulated by gene mutations. That means that there might be some other detoxification enzymes could not be effectively selected just as those in susceptible insects. These detoxification enzyme genes without mutations to upregulate could be candidates during insecticide resistance evolution. It should be taken into consideration when insecticide resistance risk evaluation and managements are performed.

Cytochrome P450 Monooxygenases (CYPs) constitute a large multi‐gene super‐family found in virtually all aerobic organisms, including organisms as diverse as insects, plants, mammals, birds, and bacteria (Stegeman & Livingstone, 1998). Insect CYPs are the most important groups of environmental response genes that play a vital role in the interactions of insects with insecticides and host plants (Wen, Zhang, & Zhang, 2011) and focused primarily on the metabolism of xenobiotics (Scott, 2008). A remarkable feature of CYPs is the large variation in substrate specificity of different CYPs (Scott, 1999). Therefore, some CYPs are capable of metabolizing a very wide range of compounds whereas some are limited to a highly restricted set of reactions (Kulkarni & Hodgson, 1980). For example, in human, CYP1A1 can metabolize more than 20 substrates, while CYP7A1 has only one known substrate and CYP2C have overlapping substrate specificity (Rendic & Carlo, 1997). In insects, though some CYPs were reported only one insecticide as a substrate, it has been confirmed that the individual CYPs can metabolize multiple insecticides and CYPs from the same species can have overlapping substrates. For example, CYP6B8, CYP6B27, and CYP321A1 from *Helicoverpa zea* can all detoxify insecticides from different classes: aldrin (cyclodiene), diazinon (organophosphate), carbaryl (carbamate), and α–cypermethrin (pyrethroid) (Li, Baudry, Berenbaum, & Schuler, 2004; Rupasinghe, Wen, Chiu, & Schuler, 2007; Sasabe, Wen, Berenbaum, & Schuler, 2004; Wen, Zeng, Niu, Berenbaum, & Schuler, 2009). Here, our experiments with the recombinant expressed protein in Sf9 cells confirmed that CYP417A2v2 from *L. striatellus* could degrade at least imidacloprid. More experiments are needed for declaring if this CYP can degrade some other insecticides or their metabolites.

On the other hand, CYPs from the same family shear higher similarity not only in gene sequence but also in function. In insects, members of the CYP family 4, 6, 9, and 12 have all been involved in detoxifying functions and among these families, the members of the CYP4 and CYP6 groups are most commonly implicated in metabolism and resistance to xenobiotics (Feyereisen, 2005; Li, Schuler, & Berenbaum, 2007). Previous research has documented that some members of the CYP4 family were highly expressed in insects and demonstrated their ability to metabolize a diverse synthetic insecticide. For example, CYP49A1 expressed in *Bombyx mori* to metabolize phoxim (Li et al., 2015), Cyp4BN13v1, and Cyp4BN15 were highly expressed in *Leptinotarsa decemlineata* larvae and involved in cyhalothrin detoxification (Wan et al., 2013) and CYP4 genes in *Diaphorina citri* are associated with the development of insecticide resistance (Killiny, Hajeri, Tiwari, Gowda, & Stelinski, 2014). For imidacloprid metabolism, previous work has demonstrated that several CYPs in different insect species, such as CYP6A1 in *N. lugens* (Ding et al., 2013), CYP6CM1vQ in *B. tabaci* (Karunker et al., 2009), and CYP6G1 in *D. melanogaster* (Joußen et al., 2008) have degrading capability. Most of them are resistance associating and from family 6. In our laboratory, the resistance associating CYPs from family 6 has also been confirmed for degrading imidacloprid in *L. striatellus*. Here we present CYP417A2v2, a resistance irrelevant gene from family 4, which should provide a good material for the study of the enzyme feature to catalyzing imidacloprid.

Otherwise, model probe chemicals were usually used to evaluate enzyme activity like CYPs and even insecticide resistance. Our results demonstrated that recombinantly expressed CYP417A2v2 have good O‐demethylation activity against PNOD and specific activities against fluorogenic substrate ethoxycoumarin. However, no activity on another fluorogenic substrate ethoxyresorufin was found. This implies that not all the traditionally used CYP activity probe substrates could use for a special CYP enzyme. For CYP417A2v2 activity, PNOD and ethoxycoumarin could be used as probing substrates, but not for ethoxyresorufin.

Finally, it can be said that resistant irrelevant CYPs for degrading insecticides provide a new insight which is opposite to the conventional idea on CYPs that are usually overexpressed and involved in insecticides degradation. Therefore, it can be concluded that detoxification CYP enzymes for a special insecticide will not be all selected during insecticide treatment and overexpressed in the resistant insect strain. We believe that some resistance irrelevant CYPs with detoxification capability could be important for insecticide resistance development, and should be taken into consideration in insecticide resistance prediction and pest management.

## CONFLICT OF INTEREST

None declared.

## References

[ece33047-bib-0501] Andersen, J. F. , Utermohlen, J. G. , & Feyereisen, R. (1994). Expression of housefly CYP6A1 and NADPH‐cytochrome P450 reductase in *Escherichia coli* and reconstitution of an insecticide‐metabolizing P450 system. Biochemistry, 33(8), 2171–2177. 8117673 10.1021/bi00174a025

[ece33047-bib-0001] Agosin, M. (1985). Role of microsomal oxidations in insecticide degradation. Comprehensive Insect Physiology, Biochemistry and Pharmacology, 12, 647–712.

[ece33047-bib-0002] Bergé, J. , Feyereisen, R. , & Amichot, M. (1998). Cytochrome P450 monooxygenases and insecticide resistance in insects. Philosophical Transactions of the Royal Society of London B: Biological Sciences, 353(1376), 1701–1705. 10021770 10.1098/rstb.1998.0321 PMC1692400

[ece33047-bib-0003] Bradford, M. M. (1976). A rapid and sensitive method for the quantitation of microgram quantities of protein utilizing the principle of protein‐dye binding. Analytical Biochemistry, 72(1–2), 248–254. 942051 10.1016/0003-2697(76)90527-3

[ece33047-bib-0004] Burke, M. D. , Thompson, S. , Weaver, R. J. , Wolf, C. R. , & Mayers, R. T. (1994). Cytochrome P450 specificities of alkoxyresorufin O‐dealkylation in human and rat liver. Biochemical Pharmacology, 48(5), 923–936. 8093105 10.1016/0006-2952(94)90363-8

[ece33047-bib-0005] Cohen, L. H. , Remley, M. J. , Raunig, D. , & Vaz, A. D. (2003). In vitro drug interactions of cytochrome p450: An evaluation of fluorogenic to conventional substrates. Drug Metabolism and Disposition, 31(8), 1005–1015. 12867489 10.1124/dmd.31.8.1005

[ece33047-bib-0006] Ding, Z. , Wen, Y. , Yang, B. , Zhang, Y. , Liu, S. , Liu, Z. , & Han, Z. (2013). Biochemical mechanisms of imidacloprid resistance in *Nilaparvata lugens:* Over‐expression of cytochrome P450 CYP6AY1. Insect Biochemistry and Molecular Biology, 43(11), 1021–1027. 23994173 10.1016/j.ibmb.2013.08.005

[ece33047-bib-0007] Elzaki, M. E. A. , Zhang, W. , Feng, A. , Qiou, X. , Zhao, W. , & Han, Z. (2015). Constitutive overexpression of cytochrome P450 associated with imidacloprid resistance in *Laodelphax striatellus* (Fallén). Pest Management Science, 72(5), 1051–1058. 26395964 10.1002/ps.4155

[ece33047-bib-0502] Elzaki, M. E. A. , Miah, M. A. , Wu, M. , Zhang, H. , Pu, J. , Jiang, L. , & Han, Z. (2017). Imidacloprid is degraded by CYP353D1v2, a cytochrome P450 over‐expressed in resistant strain of *Laodelphax striatellus* . Pest Management Science. https://doi.org/10.1002/ps.4570 10.1002/ps.4570 28296046

[ece33047-bib-0008] Feyereisen, R. (2005). Insect cytochrome P450. Comprehensive Molecular Insect Science, 4, 1–77.

[ece33047-bib-0009] Gao, B. , Wu, J. , Huang, S. , Mu, L. , & Han, Z. (2008). Insecticide resistance in field populations of *Laodelphax striatellus* Fallén (Homoptera: Delphacidae) in China and its possible mechanisms. International Journal of Pest Management, 54(1), 13–19.

[ece33047-bib-0010] Gu, B. , Xue, P. , Shi, W. , & Zhou, L. (2005). Observation on rice spikes infected by *Laodelphax striatellus* and rice yield loss. Chinese Journal of Plant Protection, 5, 002.

[ece33047-bib-0011] Guzov, V. M. , Unnithan, G. C. , Chernogolov, A. A. , & Feyereisen, R. (1998). CYP12A1, a mitochondrial cytochrome P450 from the house fly. Archives of Biochemistry and Biophysics, 359(2), 231–240. 9808765 10.1006/abbi.1998.0901

[ece33047-bib-0012] Hatano, R. , & Scott, J. (1993). Anti‐P450 lpr antiserum inhibits the activation of chlorpyrifos to chlorpyrifos oxon in house fly microsomes. Pesticide Biochemistry and Physiology, 45(3), 228–233.

[ece33047-bib-0013] Hodgson, E. , & Levi, P. E. (2010). A textbook of modern toxicology. New york: Wiley Online Library.

[ece33047-bib-0014] Inceoglu, A. , Waite, T. , Christiansen, J. , McAbee, R. , Kamita, S. , Hammock, B. D. , & Cornel, A. (2009). A rapid luminescent assay for measuring cytochrome P450 activity in individual larval *Culex pipiens* complex mosquitoes (Diptera: Culicidae). Journal of Medical Entomology, 46(1), 83–92. 19198521 10.1603/033.046.0111 PMC3522461

[ece33047-bib-0015] Joußen, N. , Heckel, D. G. , Haas, M. , Schuphan, I. , & Schmidt, B. (2008). Metabolism of imidacloprid and DDT by P450 CYP6G1 expressed in cell cultures of Nicotiana tabacum suggests detoxification of these insecticides in Cyp6 g1‐overexpressing strains of *Drosophila melanogaster*, leading to resistance. Pest Management Science, 64(1), 65–73. 17912692 10.1002/ps.1472

[ece33047-bib-0016] Karunker, I. , Morou, E. , Nikou, D. , Nauen, R. , Sertchook, R. , Stevenson, B. J. , … Vontas, J. (2009). Structural model and functional characterization of the *Bemisia tabaci* CYP6CM1vQ, a cytochrome P450 associated with high levels of imidacloprid resistance. Insect Biochemistry and Molecular Biology, 39(10), 697–706. 19716416 10.1016/j.ibmb.2009.08.006

[ece33047-bib-0017] Killiny, N. , Hajeri, S. , Tiwari, S. , Gowda, S. , & Stelinski, L. L. (2014). Double‐stranded RNA uptake through topical application, mediates silencing of five CYP4 genes and suppresses insecticide resistance in *Diaphorina citri* . PLoS One, 9(10), e110536. 25330026 10.1371/journal.pone.0110536 PMC4203802

[ece33047-bib-0018] Kisimoto, R. (1967). Genetic variation in the ability of a planthopper vector; *Laodelphax striatellus* (Fallen) to acquire the rice stripe virus. Virology, 32(1), 144–152. 6025062 10.1016/0042-6822(67)90262-0

[ece33047-bib-0019] Korytko, P. J. , & Scott, J. G. (1998). CYP6D1 protects thoracic ganglia of houseflies from the neurotoxic insecticide cypermethrin. Archives of Insect Biochemistry and Physiology, 37(1), 57–63. 9397514 10.1002/(SICI)1520-6327(1998)37:1<57::AID-ARCH7>3.0.CO;2-S

[ece33047-bib-0020] Kulkarni, A. P. , & Hodgson, E. (1980). Metabolism of insecticides by mixed function oxidase systems. Pharmacology & Therapeutics, 8(2), 379–475. 6992159 10.1016/0163-7258(80)90054-6

[ece33047-bib-0021] Laemmli, U. K. (1970). Cleavage of structural proteins during the assembly of the head of bacteriophage T4. Nature, 227, 680–685. 5432063 10.1038/227680a0

[ece33047-bib-0022] Li, X. , Baudry, J. , Berenbaum, M. R. , & Schuler, M. A. (2004). Structural and functional divergence of insect CYP6B proteins: From specialist to generalist cytochrome P450. Proceedings of the National Academy of Sciences of the United States of America, 101(9), 2939–2944. 14981232 10.1073/pnas.0308691101 PMC365724

[ece33047-bib-0023] Li, F. , Ni, M. , Zhang, H. , Wang, B. , Xu, K. , Tian, J. , … Li, B. (2015). Expression profile analysis of silkworm P450 family genes after phoxim induction. Pesticide Biochemistry and Physiology, 122, 103–109. 26071814 10.1016/j.pestbp.2014.12.013

[ece33047-bib-0024] Li, X. , Schuler, M. A. , & Berenbaum, M. R. (2007). Molecular mechanisms of metabolic resistance to synthetic and natural xenobiotics. Annual Review of Entomology, 52, 231–253. 10.1146/annurev.ento.51.110104.151104 16925478

[ece33047-bib-0025] Liu, N. , Li, M. , Gong, Y. , Liu, F. , & Li, T. (2015). Cytochrome P450s–Their expression, regulation, and role in insecticide resistance. Pesticide Biochemistry and Physiology, 120, 77–81. 25987224 10.1016/j.pestbp.2015.01.006

[ece33047-bib-0026] Liu, X. , Zhai, B. , & Liu, C. (2006). Outbreak reasons of *Laodelphax striatellus* population. Chinese Bulletin of Entomology, 43(2), 141–146.

[ece33047-bib-0027] Ma, C. , Gao, C. , Wei, H. , & Shen, J. (2007). Resistance and susceptibility to several groups of insecticides in the small brown planthopper, *Laodelphax striatellus* Homoptera. Delphacidae Chinese Journal of Rice Science, 21(5), 555–558.

[ece33047-bib-0028] Matsumura M., Otuka A., & Watanabe T., editors (2006). Migration prediction and monitoring of rice Planthoppers in Japan. Proceedings of the international symposium on area‐wide management of insect pests food and ferertilizer technology Center Okinawa, Japan.

[ece33047-bib-0029] Nakao, T. , Kawase, A. , Kinoshita, A. , Abe, R. , Hama, M. , Kawahara, N. , & Hirase, K. (2011). The A2′ N mutation of the RDL γ‐aminobutyric acid receptor conferring fipronil resistance in *Laodelphax striatellus* (Hemiptera: Delphacidae). Journal of Economic Entomology, 104(2), 646–652. 21510217 10.1603/ec10391

[ece33047-bib-0030] Nauen, R. , Vontas, J. , Kaussmann, M. , & Wölfel, K. (2013). Pymetrozine is hydroxylated by CYP6CM1, a cytochrome P450 conferring neonicotinoid resistance in *Bemisia tabaci* . Pest Management Science, 69(4), 457–461. 23325724 10.1002/ps.3460

[ece33047-bib-0031] Omura, T. , & Sato, R. (1964). The carbon monoxide‐binding pigment of liver microsomes I. Evidence for its hemoprotein nature. Journal of Biological Chemistry, 239(7), 2370–2378. 14209971

[ece33047-bib-0503] Phillips, I. , & Shephard, E. (2005). Cytochrome P450 Protocols In Methods in MolecularBiology (second ed.), 320. Totowa, NJ: Humana Press.

[ece33047-bib-0032] Pritchard, M. P. , Glancey, M. J. , Blake, J. A. , Gilham, D. E. , Burchell, B. , Wolf, C. R. , & Friedberg, T. (1998). Functional co‐expression of CYP2D6 and human NADPHcytochrome P450 reductase in *Escherichia coli* . Pharmacogenetics and Genomics, 8(1), 33–42. 10.1097/00008571-199802000-00005 9511179

[ece33047-bib-0033] Rauch, N. , & Nauen, R. (2003). Identification of biochemical markers linked to neonicotinoid cross resistance in *Bemisia tabaci* (Hemiptera: Aleyrodidae). Archives of Insect Biochemistry and Physiology, 54(4), 165–176. 14635178 10.1002/arch.10114

[ece33047-bib-0034] Rendic, S. , & Carlo, F. J. D. (1997). Human cytochrome P450 enzymes: A status report summarizing their reactions, substrates, inducers, and inhibitors. Drug Metabolism Reviews, 29(1–2), 413–580. 9187528 10.3109/03602539709037591

[ece33047-bib-0035] Riga, M. , Tsakireli, D. , Ilias, A. , Morou, E. , Myridakis, A. , Stephanou, E. , … Paine, M. (2014). Abamectin is metabolized by CYP392A16, a cytochrome P450 associated with high levels of acaricide resistance in *Tetranychus urticae* . Insect Biochemistry and Molecular Biology, 46, 43–53. 24463358 10.1016/j.ibmb.2014.01.006

[ece33047-bib-0036] Rupasinghe, S. G. , Wen, Z. , Chiu, T.‐L. , & Schuler, M. A. (2007). *Helicoverpa zea* CYP6B8 and CYP321A1: Different molecular solutions to the problem of metabolizing plant toxins and insecticides. Protein Engineering Design and Selection, 20(12), 615–624. 10.1093/protein/gzm063 18065401

[ece33047-bib-0504] Sabourault, C. , Guzov, V. , Koener, J. , Claudianos, C. , Plapp, F. , & Feyereisen, R. (2001). Overproduction of a P450 that metabolizes diazinon is linked to a loss‐of‐function in the chromosome 2 ali‐esterase (MdαE7) gene in resistant house flies. Insect Molecular Biology, 10(6), 609–618. 11903631 10.1046/j.0962-1075.2001.00303.x

[ece33047-bib-0037] Sasabe, M. , Wen, Z. , Berenbaum, M. R. , & Schuler, M. A. (2004). Molecular analysis of CYP321A1, a novel cytochrome P450 involved in metabolism of plant allelochemicals (furanocoumarins) and insecticides (cypermethrin) in *Helicoverpa zea* . Gene, 338(2), 163–175. 15315820 10.1016/j.gene.2004.04.028

[ece33047-bib-0038] Schulz‐Jander, D. A. , & Casida, J. E. (2002). Imidacloprid insecticide metabolism: Human cytochrome P450 isozymes differ in selectivity for imidazolidine oxidation versus nitroimine reduction. Toxicology Letters, 132(1), 65–70. 12084621 10.1016/s0378-4274(02)00068-1

[ece33047-bib-0039] Scott, J. G. (1999). Cytochromes P450 and insecticide resistance. Insect Biochemistry and Molecular Biology, 29(9), 757–777. 10510498 10.1016/s0965-1748(99)00038-7

[ece33047-bib-0040] Scott, J. G. (2008). Insect cytochrome P450s: Thinking beyond detoxification. Recent Advances in Insect Physiology, Toxicology and Molecular Biology, 1, 17–124.

[ece33047-bib-0041] Stegeman, J. , & Livingstone, D. (1998). Forms and functions of cytochrome P450. Comparative biochemistry and physiology Part C. Pharmacology, Toxicology & Endocrinology, 121(1–3), 1–3. 9972446

[ece33047-bib-0042] Taylor, M. , & Feyereisen, R. (1996). Molecular biology and evolution of resistance of toxicants. Molecular Biology and Evolution, 13(6), 719–734. 8754209 10.1093/oxfordjournals.molbev.a025633

[ece33047-bib-0043] Wan, P. , Shi, X. , Kong, Y. , Zhou, L. , Guo, W. , Ahmat, T. , & Li, G. (2013). Identification of cytochrome P450 monooxygenase genes and their expression profiles in cyhalothrin‐treated Colorado potato beetle, *Leptinotarsa decemlineata* . Pesticide Biochemistry and Physiology, 107(3), 360–368. 24267698 10.1016/j.pestbp.2013.10.004

[ece33047-bib-0044] Wang, H. , Chen, J. , Zhang, H. , Sun, X. , Zhu, J. , Wang, A. , … Adams, M. (2008). Recent rice stripe virus epidemics in Zhejiang province, China, and experiments on sowing date, disease–yield loss relationships, and seedling susceptibility. Plant Disease, 92(8), 1190–1196. 10.1094/PDIS-92-8-1190 30769483

[ece33047-bib-0054] Wang, Y. , Wu, C. , Zhao, X. , Cang, T. , Chen, L. , Yu, R. , … Wang, Q. (2010). Advances in the research of insecticide resistance of the small brown planthopper, *Laodelphax striatellus* . Plant Protection, 4, 009.

[ece33047-bib-0045] Wang, L. , Zhang, Y. , Han, Z. , Liu, Y. , & Fang, J. (2010). Cross‐resistance and possible mechanisms of chlorpyrifos resistance in *Laodelphax striatellus* (Fallén). Pest Management Science, 66(10), 1096–1100. 20582994 10.1002/ps.1984

[ece33047-bib-0046] Wei, L. (2007). Research progress of sustainable controls and ecological characteristics of *Laodelphax striatellus* . Journal of Anhui Agricultural Sciences, 35(8), 2323.

[ece33047-bib-0047] Wen, Z. , Zeng, R. S. , Niu, G. , Berenbaum, M. R. , & Schuler, M. A. (2009). Ecological significance of induction of broad‐substrate cytochrome P450s by natural and synthetic inducers in Helicoverpa zea. Journal of Chemical Ecology, 35(2), 183–189. 19198946 10.1007/s10886-009-9598-4

[ece33047-bib-0048] Wen, Z. , Zhang, X. , & Zhang, Y. (2011). P450—mediated insecticide detoxification and its implication in insecticide efficacy. Recent advances in entomological research (pp. 229–245). Berlin Heidelberg: Springer.

[ece33047-bib-0049] Wheelock, G. D. , & Scott, J. G. (1992). The role of cytochrome P450_lpr_ in deltamethrin metabolism by pyrethroid‐resistant and susceptible strains of house flies. Pesticide Biochemistry and Physiology, 43(1), 67–77.

[ece33047-bib-0050] Wilkinson, C. , & Brattsten, L. (1972). Microsomal drug metabolizing enzymes in insects. Drug Metabolism Reviews, 1(1), 153–227.

[ece33047-bib-0051] Xu, L. , Wu, M. , & Han, Z. (2013). Overexpression of multiple detoxification genes in deltamethrin resistant *Laodelphax striatellus* (Hemiptera: Delphacidae) in China. PLoS One, 8(11), e79443. 24324548 10.1371/journal.pone.0079443 PMC3855578

[ece33047-bib-0052] Yang, Y. , Wu, Y. , Chen, S. , Devine, G. , Denholm, I. , Jewess, P. , & Moores, G. (2004). The involvement of microsomal oxidases in pyrethroid resistance in *Helicoverpa armigera* from Asia. Insect Biochemistry and Molecular Biology, 34(8), 763–773. 15262281 10.1016/j.ibmb.2004.04.001

[ece33047-bib-0053] Yang, Y. , Yue, L. , Chen, S. , & Wu, Y. (2008). Functional expression of *Helicoverpa armigera* CYP9A12 and CYP9A14 in *Saccharomyces cerevisiae* . Pesticide Biochemistry and Physiology, 92(2), 101–105.

[ece33047-bib-0055] Zhang, X. , Chen, J. , Chen, L. , & Yu, X. (2007). Resistance monitoring of the smaller brown plathopper, *Laodelphax striatellus* fallen to imidacloprid, fipronil and chlorpyriphos in Zhejiang province. Acta Agriculturae Zhejiangensis, 19(6), 435.

[ece33047-bib-0056] Zhang, Y. , Guo, H. , Yang, Q. , Li, S. , Wang, L. , Zhang, G. , & Fang, J. (2012). Overexpression of a P450 gene (CYP6CW1) in buprofezin‐resistant *Laodelphax striatellus* (Fallén). Pesticide Biochemistry and Physiology, 104(3), 277–282.

[ece33047-bib-0057] Zhang, M. , & Scott, J. G. (1996). Cytochrome b 5 is essential for cytochrome P450 6D1‐mediated cypermethrin resistance in LPR house flies. Pesticide Biochemistry and Physiology, 55(2), 150–156. 8980039 10.1006/pest.1996.0044

[ece33047-bib-0058] Zimmer, C. T. , Bass, C. , Williamson, M. S. , Kaussmann, M. , Wölfel, K. , Gutbrod, O. , & Nauen, R. (2014). Molecular and functional characterization of CYP6BQ23, a cytochrome P450 conferring resistance to pyrethroids in European populations of pollen beetle, *Meligethes aeneus* . Insect Biochemistry and Molecular Biology, 45, 18–29. 24316412 10.1016/j.ibmb.2013.11.008

